# Autonomic nervous system responses to strength training in top‐level weight lifters

**DOI:** 10.14814/phy2.14233

**Published:** 2019-10-22

**Authors:** Ferdinando Iellamo, Daniela Lucini, Maurizio Volterrani, Maurizio Casasco, Annamaria Salvati, Antonio Gianfelici, Alessia Di Gianfrancesco, Antonio Urso, Vincenzo Manzi

**Affiliations:** ^1^ Department of Clinical Science and Translational Medicine and School of Sports Medicine University Tor Vergata Rome Italy; ^2^ Scientific Institute of Research and Scientific Institute of Research and Care Care San Raffaele Pisana Rome Italy; ^3^ BIOMETRA Exercise Medicine Unit University of Milan Humanitas Milan Italy; ^4^ Italian Federation of Sport Medicine Rome Italy; ^5^ Italian Federation of weight lifting Rome Italy; ^6^ University Foro Italico Rome Italy

**Keywords:** Heart rate variability, strength training, training adaptations: training monitoring, weight lifters

## Abstract

In athletes, spectral analysis of HR variability (HRV) has been shown capable to detect the adaptational changes in sympatho‐vagal control attending physical training. So far, studies investigated autonomic nervous system (ANS) changes occurring with endurance training, whereas adaptations to markedly different exercise modes, for example, strength training, have never been investigated. We assessed the changes in cardiac ANS parameters during long‐term training in weight lifters of the Italian team preparing for the European Championship, where athletes competed for obtaining the pass for Olympic Games. We investigated nine athletes. Subject trained 3 sessions/day, 6 days a week. The intensity of strength exercises varied from 70% to 95% 1 RM. Training load (TL) was calculated as: volume (min) × intensity (%1RM).All ANS parameters were significantly and highly correlated on an individual basis to the dose of exercise with a second‐order regression model (*r* ^2^ ranged from 0.96 to 0.99; *P* < 0.001). The low‐frequency (LF) component of HRV and LF/HF ratio showed an initial increase with the progression of TL and then a decrease, resembling a bell‐shaped curve with a minimum at the highest TL. The high‐frequency (HF) component of HRV and R‐R interval showed a reciprocal pattern, with an initial decrease with progression of TL followed by an increase, resembling an U‐shaped curve with a maximum at the highest TL. These adaptations were at the opposite to those previously reported in endurance athletes. These results suggest that in Olympic weight lifters, ANS adaptations to training are dose‐related on individual basis and that ANS adaptations are mainly sport‐specific.

## Introduction

Searching for minimally invasive, minimally disturbing indicators of training status in athletes has been always a matter of interest in exercise physiology and sports medicine. To this aim, several variables have been monitored, mostly related to adaptive changes in the neuroendocrine system and particularly heart rate (HR), because HR represents one of the most accessible, noninvasive and low‐cost physiological measures in sports medicine. In this context, spectral analysis of short‐term HR variability (HRV) has been shown to be capable to detect the complex adaptational changes in sympatho‐vagal control attending physical training.

Indeed, we and other groups reported the feasibility and reliability of HRV in monitoring autonomic nervous system (ANS) changes with training in healthy subjects (Iwasaki et al. 2003; Okazaki et al. 2005) cardiac patients (Iellamo et al. 2013) and athletes (Iellamo et al. 2002, 2004; Manzi et al. 2009).

However, most of the studies performed so far, investigated ANS changes occurring with endurance, aerobic training, and showed a dose–response relationship of ANS responses to training, best described by a second‐order regression model (Iwasaki et al. 2003; Okazaki et al. 2005; Manzi et al. 2009; Iellamo et al. 2013) with different and reciprocal shapes for parasympathetic and sympathetic indicators.

Aerobic exercises consist of activities performed for prolonged periods that involve large muscles masses (e.g., running and cycling). Aerobic exercise induces many physiological adaptations, mediated at both central and peripheral sites (Wilmore et al. 2008). The main metabolic adaptations to aerobic exercise at muscular level are a slower consumption of muscle glycogen, a larger reliance on fat oxidation, and less lactate production during exercise at a given intensity (Wilmore et al. 2008).

To what extent adaptations in ANS regulation observed in endurance athletes extend to markedly different exercise training modes, for example, strength training, has been much less investigated.

Strength training is a type of physical exercise that provides meaningful functional benefits to the health and athletic performance, including muscle hypertrophy and, possibly, hyperplasia (Folland and Williams 2007; Roberts et al. 2015). Traditionally strength training programs consist of exercises with resistance or added weight, comprising repetitions before muscle exhaustion and in weight lifters include to a large extent, olympic lifts (snatch, clean and jerk), powerlifts etc (see Methods).

To the best of our knowledge, no study has analyzed the relationship between training load and ANS parameters in high‐level strength‐trained athletes.

In the present investigation, we assessed the changes in cardiac ANS parameters with training load in weight lifters of the national Italian team preparing for the European Championship 2016 and tested the hypothesis that changes in ANS with weight‐lifting‐specific training are different from those described with endurance training, being therefore sport‐specific.

## Methods

This study was conducted on the entire group of weight lift athletes of the National Italian Team over the season culminating with the Rio de Janeiro 2016 Olympic Games. All athletes had been previously screened for cardiovascular or metabolic diseases that could contraindicate participation in agonistic competitions.

### Subjects

Nine healthy, trained, weight lifters (5 males and 4 females, age 20 to 39 years, of weight class from 48 Kg to 70 Kg, with at least 6 years of high‐level competitions (all had participated to international competitions and medal winners) volunteered to participate in the study. All subjects provided informed written consent to the experimental procedures after the possible benefits and risks of participation were explained to them. The study protocol was approved by Institutional Review Board of Sports Medicine Institute CONI and followed the guidelines laid down by the World Medical Assembly Declaration of Helsinki.

#### Experimental protocol

Before the beginning of the study, all the athletes abstained from vigorous efforts for 4 weeks to avoid possible effects over the experimental intervention; thus, for the purpose of this study, at this time they were considered as (partially) detrained and underwent the baseline recording sessions. Thereafter, each athlete was investigated on three subsequent occasions during the season, according to the training periodization by the coach. The last assessment was performed just before the European Championship 2016, where athletes competed to obtain the pass for the Rio de Janeiro 2016 Olympic Games. All the recording sessions were performed early in the morning after an overnight fasting, before breakfast.

No one athlete was considered overtrained at the time of the recording sessions, based on the lack of the following signs: an inability to sustain the usual training program and the presence of symptoms, such as increased feelings of fatigue during daily training routine, sleeping disorders, apathy, or restlessness. No athlete was taking drugs at the time of the recording sessions. This was monitored by the physician’s team.

#### Training protocol and training load calculation

The subject trained about 18 times a week (3 sessions/day, 6 days a week), according to their individual program. Training routine consisted of different weight exercises: olympic lifts (snatch, clean and jerk), powerlifts, pulling exercises, and squat lifts, for a total of 90–100 repetitions per day. The average intensity of all strength exercises varied from 70% to 95% of 1‐repetition maximum (1 RM). The training parameters, that is, the volume and intensity of the different types of exercise were recorded during the whole experimental period. The training volume used in the training load calculation, was the total training time, while the intensity was the percentage of 1‐RM.

Therefore, training load (TL) was calculated as follows:

$$\text{TL}\left[\text{arbitrary units},\;\left(\text{AU}\right)\right]\;=\text{volume}\left(\text{min}\right)\;\times \text{intensity}\left(\%\text{1RM}\right).$$

The sum from all sessions for each given training cycle provided the total training load for that cycle. All sessions were supervised by the coach to monitor the appropriate amount of exercises.

#### Autonomic nervous system assessment

The continuous ECG signal was obtained with a modified C5 lead, connecting the electrodes to an analog preamplifier (BT 16 plus, Marazza, Monza, Italy). Respiratory signal was recorded with a piezoelectric thoracic belt. The analog signals were connected to an A/D board inserted in a personal computer, sampled at 250 Hz, and stored on the hard disk for subsequent analyses. These signals were used to assess autonomic function. Athletes did not perform strenuous physical activities in the 20 h before recordings. All the recordings were performed in a room at ambient temperature (22–24°C) in the Sports Medicine Institute CONI of Rome. After instrumentation, the subjects lay supine for 15 min before experiments to relax in the room made dark and noiseless; thereafter, continuous ECG data acquisition was performed for 10 min.

#### Power spectral analysis

A purposely developed software (Heartscope, ver.1.6, A.M.P.S. llc, New York) (Badilini et al. 2005) was used to identify the peak of R wave on ECG. The software constructs automatically time series of RR intervals and respiratory activity (RESP) with low operator‐analysis interaction. Spontaneous variability of RR interval and RESP was evaluated by means of power spectral analysis using an autoregressive algorithm, as previously described (Pagani et al. 1986, 1997; Manzi et al. 2009). Briefly, the harmonic components of RR interval were evaluated by the autoregressive method. Components in the frequency band from 0.03 to 0.15 Hz were considered low frequency (LF), and those in the range from 0.15 to 0.4 Hz, were considered high frequency (HF). The LF component of RR interval (when expressed in normalized units) is considered to be an expression of mainly cardiac efferent sympathetic regulation, whereas the HF component of RR interval variability is considered to be an expression of cardiac vagal modulation (Pagani et al. 1986, 1997; Task Force of the European Society of Cardiology and the North American Society of Pacing and Electrophysiology Task Force of the European Society of Cardiology and the North American Society of Pacing and Electrophysiology, 1996; Iellamo et al. 2001). Oscillations slower than 0.03 Hz were considered as very low frequency components (i.e., DC noise). Spectral analysis of the respiratory signal was performed on the signal sampled once for every cardiac cycle. Respiratory spectra were used to assess the main respiratory frequency. The power density of each spectral component was calculated both in absolute values and normalized units (n.u.), computed as the ratio of the absolute power of either HF or LF to the total power, less the very‐low‐frequency component if present, and multiplying this ratio by 100 (Pagani et al. 1986). The use of normalized units is crucial in order to obtain valuable information as to the oscillatory cardiac modulation, because of the high interindividual variability in R‐R interval total variance and DC noise (Pagani et al. 1986, 1997; Iellamo et al. 2001) and possible redundancy of indices (Lucini et al. 2018).

### Statistics

The significance of differences in the ANS parameters among the different recording sessions was evaluated by analysis of variance (ANOVA) for repeated measures. Effect size (*η* ^2^) was also calculated according to Cohen (1988) and values of 0.01, 0.06, and >0.14 were interpreted as small, medium and large, respectively. To express the dose–response relationship between the training stimulus and changes in autonomic cardiac regulation indexes, correlations between the training load and autonomic cardiac regulation indexes at baseline and at the different times during the training season were estimated from a second‐order regression, accordingly to previous studies (Iwasaki et al. 2003; Okazaki et al. 2005; Manzi et al. 2009). Differences were considered statistically significant when *P* ≤ 0.05. A commercial package (SPSS, version 20.0 for Windows; Chicago, IL) was used for all statistical calculations. The results are expressed as mean ± SEM.

## Results

**Figure 1:**
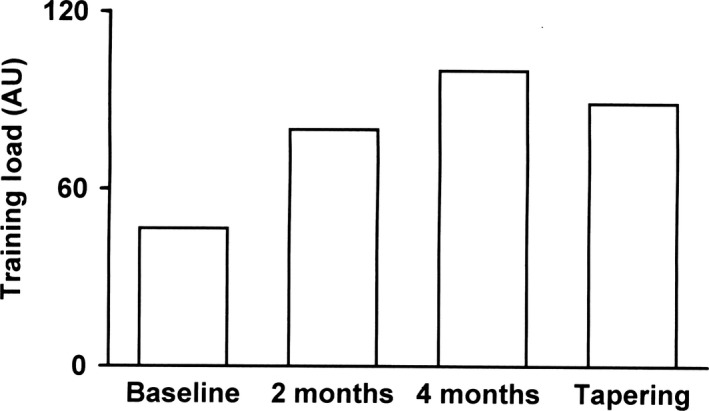
Time course changes in training load during the competitive season.

TL progressively increased during the season then decreased during the tapering period of the European Championship (Fig. 1). Baseline heart rate (HR) and systolic and diastolic arterial pressure were 62 ± 1.3 b/min, 100 ± 14 mmHg, and 65 ± 8 mmHg, respectively, and did not change significantly throughout the study, as did breathing frequency, that ranged from 0.29 to 0.27 Hz.

### Spectral analysis of HRV

**Figure 2:**
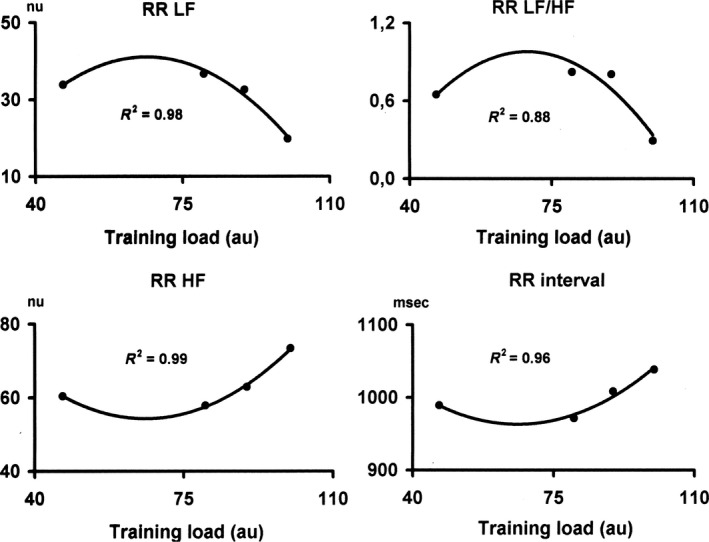
Dose–response relationship between training load and autonomic cardiac indexes. HF, High‐frequency; LF, Low‐frequency components of R‐R interval variability; LF/HF, Low‐ to High‐frequency ratio in R‐R interval variability; NU, normalized units. Entries represent mean values ± SEM for nine athletes.

The LF power of R‐R interval variability (normalized units) showed an increase with the increase in TL and then a decrease, as did the LF/HF ratio. The HF component of R‐R interval variability (normalized units) showed a reciprocal pattern, with a decrease as TL progressed, followed by an increase. The same occurred for R‐R interval. Although the mean changes at the group level in all indexes of autonomic cardiovascular regulation were not statistically significant (*P*> 0.05), the effect size calculated using partial eta squared were moderate or large (RR_Mean_, *η* ^2^ = 0.087; RR LF_nu_, *η* ^2^ = 0.226; HF_nu_, *η* ^2^ = 0.177; RR LF/HF, *η* ^2^ = 0.133). As shown in Figure 2, the ANS parameters as well as R‐R interval were significantly and very highly correlated with the dose of exercise with a second‐order regression model (*r* ^2^ ranged from 0.96 to 0.99; *P* < 0.001), with different and reciprocal shapes for parasympathetic and sympathetic indicators. HF_NU_ and R‐R interval (and total variance as well), resembled an U‐shaped curve with a maximum at the highest TL, whereas LF_NU_ and LF/HF ratio resembled a bell‐shaped curve with a minimum at the highest TL.

Of the nine athletes competing at the European Championship, four won medals that were spread across weights categories and sex. Two female athletes won a silver and a bronze medal while two male athletes won a gold and silver medal, respectively.

## Discussion

The main and novel finding of the present investigation is that cardiac ANS adaptations to strength training in top‐level weight lift athletes are dose‐related on individual basis, and are substantially different from those observed in endurance‐trained athletes, showing a progressive shift toward a parasympathetic predominance as training load approached the maximum. Hence, ANS adaptations to training in top‐level athletes appear to be mainly sport‐specific and not generalized.

### ANS adaptations to training

**Figure 3:**
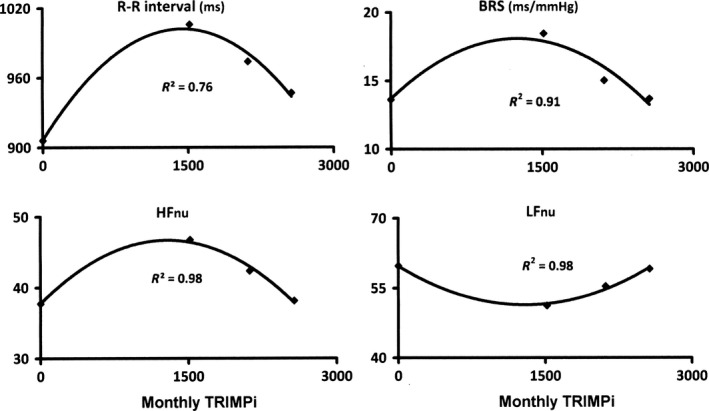
Dose–response relationship between exercise intensity/volume (monthly TRIMPi) and autonomic cardiovascular indexes. BRS, Baroreflex Sensitivity, HF, High‐frequency and LF Low‐frequency components of R‐R interval variability, NU, normalized units. Entries represent median values for eight athletes. Used by permission from Manzi et al. (2009).

We recently reported consistent data on the dependence of ANS adaptations upon TL, on an individual basis, in endurance sports (Manzi et al. 2009; Iellamo et al. 2013). Specifically, indexes of parasympathetic cardiac regulation showed a bell‐shaped curve with a minimum at the highest training load, whereas indexes of sympathetic cardiac regulation resembled an U‐shaped curve with a maximum at the highest training load (Manzi et al. 2009; Iellamo et al. 2013) (Fig. 3). As TL approached the maximum there is an increase in the LF component of HRV and in the LF/HF ratio and a decrease in the HF component of HRV and baroreflex sensitivity (BRS) (Manzi et al. 2009) (Fig. 3), in keeping with previous studies suggesting that the magnitude of training load alters cardiac autonomic modulation in a direction that would be consistent with a sympathetic predominance (Pichot et al. 2000; Portier et al. 2001; Iellamo et al. 2002, 2004).

The results of the present investigation in weight‐lifting world‐class athletes, that experience markedly different training routines in comparison to endurance‐trained athletes, are at variance with the above findings, even though they confirm the nonlinear dose–response relationship between the exercise training stimulus and dynamic regulation of HR. This concept would be supported by the finding that individual dose–response changes were detected across athletes of different sex, age and weight categories (ranging from 48 to 85 kg), hence experiencing *different absolute* TL but *the same relative* TL. Overall, it does appear that cardiac ANS adaptations are strongly dependant, on an individual basis, on the type of training being performed and, therefore, are dependent to a large extent on sport‐specific training practises. To our knowledge, this is the first study to have addressed ANS adaptations to strength training in weight‐lifting world‐class athletes during a whole season culminating with a high demanding competition.

In our study no significant differences in ANS parameters with variations in TL have been detected on a group level. The more likely explanation for the discrepancy in ANS parameters on individual versus group level, is the large inter‐individual difference in HRV parameters at baseline and throughout the study, along with weight category, age, and sex differences between athletes, which prevented the detection of significant differences in mean values with TL variations and the small sample size. This explanation would be supported by a previous study by Manzi et al (Manzi et al. 2009) showing the same occurrence, that is, discrepancy in ANS parameters on individual versus group level, in endurance athletes [and in cardiac patients as well (Iellamo et al. 2013)].

It thus appears that to adequately examine the relation between ANS and physical training, it is necessary to account for the relative degree of effort expended by each athlete individually, in addition to training specificity.

Regrettably, our results cannot be directly compared to previous investigations in top‐level athletes addressing the link between ANS changes and long‐term strength training programs in the preparation for a competition. Current knowledge regarding the link between ANS changes and weight training is lacking, since long‐term HRV‐monitored studies have mainly addressed endurance training.

As far as resistance training is concerned, available data, not obtained in elite athletes, collectively would indicate that this type of exercise does not affect resting HRV in healthy young and older individuals (Kingsley and Figueroa, 2016; Bhati et al. 2018).

A strength of the present investigation is the repeated measurement of ANS parameters during the training period, which could have improved our comprehension of the link between changes in training load and changes in cardiac autonomic regulation, although the mechanism(s) underlying this effect were not examined as a part of this study and need to be defined.

The opposite changes in ANS parameters with changes in TL between endurance (Iwasaki et al. 2003; Manzi et al. 2009; Iellamo et al. 2013) and strength training (present study) might be ascribed to differences in the single exercise routines during the training sessions, with endurance exercises requiring a more prolonged cardiac demand, implying a greater sympathetic activation, in comparison to the much shorter, although intense, requirements of weight‐lifting exercise routines.

### Limitations

The main limitation of the present investigation is the small sample size, which, unfortunately, is a common and unavoidable characteristic of studies carried out in athletes of top‐class level. Similarly, we performed a four point assessment that could be perceived as few. However, it is highly difficult to have national‐class athletes available for more frequent assessments over the whole year. On the other hand, most studies in this field performed only two assessments (i.e., before and after training) thus precluding an accurate delineation of the link between serial changes in training load and changes in cardiac ANS regulation and a dose–response analysis. The strong correlation between ANS parameters and training load (*r* ^2^ ranging from 0.89 to 0.99) also argues against a chance effect. In addition, we could not discriminate between male and female athletes, because of the small number of athletes of both sexes. Again, however, the strong consistency of our data on individual dose–response relationships would argue for sex‐independent ANS adaptations, although this point should be confirmed. Finally, the study lacks a control group that did not exercise. However, within the framework of the present investigation, this would be more a theoretical rather than an actual limitation. Indeed, it would be hard to hypothesize dose–response training‐related changes in ANS, as those observed in our study, in subjects who do not undergo exercising training. Indeed, there would be virtually no rationale to investigate the relationship between ANS adaptations and training in nonexercising individuals.

We should also mention that we used an indirect method to assess changes in autonomic function. Although this procedure stimulated strong debates in the literature (Eckberg, 1997; Malliani et al. 1998), nevertheless several studies have affirmed that spectral analysis of HRV is a simple way to extract the information embedded in the frequency code characterizing neural cardiovascular regulation (Pagani et al. 1986, 1997; Task Force of the European Society of Cardiology and the North American Society of Pacing and Electrophysiology Task Force of the European Society of Cardiology and the North American Society of Pacing and Electrophysiology, 1996; Iellamo et al. 2001). Indeed, the issue of the validity of the spectral analysis approach was addressed by experiments in humans (Pagani et al. 1997) in whom direct recordings of muscle sympathetic nerve activity were performed during various states of autonomic regulation, as produced by graded infusions of vasodilators and vasoconstrictors. The presence of similar, coherent oscillations at LF in nerve activity, R‐R intervals, and systolic arterial pressure (SAP) variabilities at various levels of induced pressure changes, provides support for the use of LF_R‐R_ (in n.u.) as an index of mainly sympathetic modulation of the sinoatrial node. The lack of LF oscillations in the R‐R interval [and SAP variability as well, which reflects vascular efferent sympathetic regulation (Task Force of the European Society of Cardiology and the North American Society of Pacing and Electrophysiology Task Force of the European Society of Cardiology and the North American Society of Pacing and Electrophysiology, 1996; Pagani et al. 1997)] in tetraplegic patients who lack the ability to modulate sympathetic nerve traffic to the heart and vasculature (Iellamo et al. 2001) provides further experimental support to the above concept.

In conclusion, the results of this study indicate that in weight‐lifting‐trained world‐class athletes there is a curvilinear dose–response relationship between training load and ANS functioning parameters, on an individual basis, as previously reported in endurance‐trained athletes. At variance with endurance‐trained athletes, however, in weight‐lifting‐trained athletes ANS adaptations go in an opposite direction with an increase in vagal and a reciprocal decrease in sympathetic indicators with the progression of training load (compare Figs. 2 and 3).

The study confirms that monitoring of HRV might have practical in addition to physiological implications, in that it could provide additional information useful to assess the dynamics of training in weightlifters during the training period and before competition through a simple, noninvasive, and minimally time‐consuming approach.

## Conflict of Interest

None declared.
